# Review of guidelines for the identification and clinical care of patients with genetic predisposition for hematological malignancies

**DOI:** 10.1007/s10689-021-00263-z

**Published:** 2021-05-31

**Authors:** Brigitte Schlegelberger, Cristina Mecucci, Marcin Wlodarski

**Affiliations:** 1 Department of Human Genetics, Hannover Medical School, Hannover, Germany; 2 Institute of Hematology and Center for Hemato-Oncology Research, University and Hospital of Perugia, Perugia, Italy; 3 Division of Pediatric Hematology and Oncology, Medical Center, Department of Pediatrics and Adolescent Medicine, Faculty of Medicine, University of Freiburg, Freiburg, Germany; 4 Department of Hematology, St. Jude Children’s Research Hospital, Memphis, TN USA

**Keywords:** Genetic predisposition, Germline, Leukemia, Hematologic malignancies

## Abstract

Since WHO has recognized myeloid neoplasms with germline predisposition as a new entity in 2016, it has become increasingly clear that diagnosing familial leukemia has critical implications for both the patient and his/her family, and that interdisciplinary teams of hematologists and clinical geneticists should provide care for this specific patient group. Here, we summarize consensus criteria for the identification and screening of patients with genetic predisposition for hematologic malignancies, as provided by different working groups, e.g. by the Nordic MDS group and the AACR. In addition to typical clinical features, results from targeted deep sequencing may point to a genetic predisposition. We review strategies to distinguish somatic and germline variants and discuss recommendations for genetic analyses aiming to identify the underlying genetic variant that should follow established quality criteria to detect both SNVs and CNVs and to determine the pathogenicity of genetic variants. To enhance the knowledge about hematologic neoplasms with germline predisposition we recommend archiving clinical and genetic data and archiving them in international registries.

## Introduction

About five to 10 percent of leukemia patients are estimated to carry a germline variant predisposing them to MDS/AML [1]. In specific situations, leukemia risk is even higher and amounts up to 70% for MDS with monosomy 7 in adolescents [2]. In addition to specific somatic chromosome aberrations such as monosomy 7, features indicative of a genetic predisposition are multiple malignancies, family history of hematological neoplasms, long-lasting thrombocytopenia, not explained by other reasons, congenital abnormalities, excessive treatment toxicities, delay count recovery after chemotherapy or poor stem cell mobilization [3, 4].

In contrast to sporadic leukemia, where only the leukemic cells carry genetic variants, in familial leukemia all cells of the body carry a germline variant. These germline variants may occur de novo*,* but in many cases, they are inherited and may be passed from one generation to the next. Most leukemia risk syndromes are inherited in an autosomal dominant fashion, with a 50% (1:2) chance for children to inherit the causative germline variant. Not every individual carrying the germline variant will develop disease, some might have no symptoms or only show mild cytopenia—a phenomenon called reduced penetrance [5]. Examples for autosomal recessive inherited diseases with high MDS/AML risk are classical bone marrow failure syndromes such as Fanconi anemia (FA) and Shwachman-Diamond syndrome, examples for X-linked inheritance are some forms of dyskeratosis congenita (*DKC1*) or FA (*FANCB*) and the immunodeficiency Wiskott-Aldrich syndrome. For these disorders, detailed guidelines and expert recommendations exist [6–9].

In 2016 WHO has recognized myeloid neoplasms with germline predisposition as a new entity (Table 1) [10, 11]. They are classified as (1) neoplasms with germline predisposition without pre-existing conditions (e.g. *CEBPA*, *DDX41*), (2) neoplasms with a history of thrombocytopenia (*RUNX1*, *ANKRD26*, *ETV6*) and (3) neoplasms with other organ dysfunction (e.g. *GATA2* or telomere biology disorders).

**Table 1:** Comparison of the Nordic, the Spanish and the guidelines of the American Association for Cancer Research (AACR)

	Nordic guidelines [20]	Spanish guidelines [23]	AACR guidelines [21]
Focus	Adult patients	Targeted deep sequencing in adult patients with MDS/ CMML	Pediatric patients
Provision of clinical care	Interdisciplinary team of hemato-oncologists and clinical geneticist	Professionals with expertise in cancer predisposing syndromes and in genetic counselling	Centers with expertise in hereditary hematologic malignancies including hematologists-oncologists and geneticists
Pre-conditions of genetic testing	Genetic counselling for *all* patients investigated for germline variants, also if no pathogenetic variant is detected	Testing for somatic mutations using myeloid panel plus recommended panel to identify germline variants such as *DDX41* on bone marrow derived cells	Pre- and post-test genetic counselling *for siblings and parents* to exclude mutation carriers as stem cell donors
Criteria for whom to test			
Family history	Patients with positive family history or symptoms indicative of a hereditary condition predisposing to myeloid neoplasms before the age of 50*		
Somatic variants	VAF 40–60% (near-heterozygous) or > 90% (near-homozygous) Patients carrying monosomy 7/del(7q)/der(7) before the age of 50		Variant allele frequency close to 50 or 100%
Investigations at baseline	CBC Bone marrow aspirate with cytogenetic analysis and testing for somatic mutations using myeloid panel		CBC Bone marrow aspirate with morphology and cytogenetic analysis Physical examination Family history Medical history (especially prior cytopenias, bleeding history)
Follow up			
CBC	Every 6 months		Once a year (morphology and cytogenetics)
Bone marrow aspirate	Only in case of change in CBC		Once a year (morphology and cytogenetics) for those at higher risk of MDS/AML even with stable blood counts, not necessary for asymptomatic children with stable blood count and lower risk of MDS/AML
NGS myeloid panel of blood	Once a year		
Surveillance of other organ dysfunctions			

*A Patients with positive family history or signs/symptoms indicative of a hereditary condition predisposing to myeloid neoplasms (MN) especially MDS and AML. *A1* Patient with MDS or AML and symptoms/signs of a hereditary condition predisposing to MN development diagnosed before the age of 50. *A2* Two individuals (first or second-degree relatives, FDR, and SDR, respectively) with MDS or AML or long-lasting thrombocytopenia or symptoms/signs indicative of a hereditary condition predisposing to MN development, one of whom diagnosed before the age of 50. *A3* One individual with MDS or AML and two FDR or SDR with a diagnosis of solid tumor malignancy one of whom diagnosed before the age of 50. *B* Patients with MN where the diagnostic work-up for the determination of the somatic genomic background has detected gene variants suspected to be germline (near heterozygous or near homozygous). *C* Patients not fulfilling the criteria A and B diagnosed with MDS or AML before the age of 50 carrying aberrations of chromosome 7 [monosomy 7/del(7q)/der(7)]

**Fig. 1:**
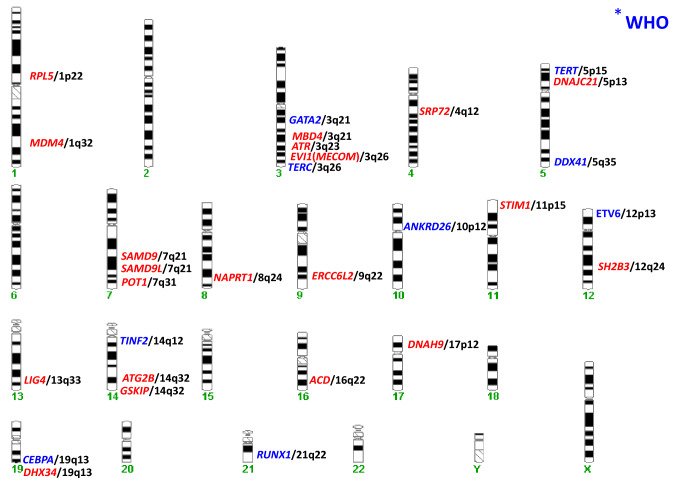
Genes associated with germline predisposition for hematologic neoplasms and their chromosomal location. Blue: genes recognized as myeloid neoplasms with germ line predisposition by the WHO in 2016. Red: genes published afterwards

In the meantime, a number of additional risk gene loci have been identified (Fig. 1) [12]. It has become clear, that the spectrum of hematologic malignancies in certain genetic tumor risk syndromes contains myeloid, but also lymphoid neoplasms [13, 14]. In Li-Fraumeni syndrome, typical leukemia subtypes are MDS/ AML with complex karyotypes, but also hypodiploid acute lymphoblastic leukemia (ALL) [15]. In *RUNX1-*familial platelet disorder with myeloid malignancies (FPDMM), MDS/ AML, but also T-ALL may occur [16]. Also *ETV6-*associated familial leukemias comprise both myeloid and lymphoid neoplasms [17]. For patients with pathogenic germline variants in *PAX5* [18–20] and *IKZF1* [21], lymphoid leukemias have been reported. Infection stimuli may play a role in leukemogenesis in predisposed individuals [22].

A recent study performed in a cohort of 86 families with MDS/AML established germline variants with a high or moderate level of evidence for gene-disease association in 16 genes previously associated with malignancies in 57% (49 families) [23]. In a further uncharacterized 37 families 65 new candidate loci were identified. These included genes that are known to be mutated in inherited hematological disorders, including *ADA*, *GP6*, *IL17RA*, *PRF1*, *SEC23B*, *DNAH9*, *NAPRT1*, *SH2B3* or novel loci, e.g. *DHX34*. Notably, the most frequently affected gene was *RUNX1, w*hich was mutated in 12% of the patients. In addition to single nucleotide variants (SNVs), truncating and splice site variants, a significant number of copy number variants such as complete *RUNX1* deletions were detected. This has to be considered when selecting methods for the identification of genetic variants [24].

## Consensus criteria for the identification of patients with genetic predisposition for hematologic malignancies

Since the diagnosis of familial leukemia has critical implications for the patient and for his/her family, it is important to recognize clinical features pointing towards genetic predisposition [1]. In 2016 Jongmans et al. have described easy to use recognition criteria of genetic predisposition in pediatric cancer patients [25]. They include (1) family history, (2) specific cancer entities, (3) somatic hints, (4) ≥ 2 malignancies, (5) congenital defects/ non-malignant signposts, (6) high/unexpected toxicity. These criteria have been updated by the German Societies of Paediatric Hematology and Oncology and Human Genetics in a questionnaire designed for use in every pediatric hematology oncology center [26]. It is requested to take a 3-generation pedigree to evaluate the family history, to document diagnosis of neoplasms or known germline predisposition determined by genetic tumor analysis, two or more known malignancies or the presence of a child with cancer and congenital or other anomalies, and, finally, whether the patient has suffered from excessive toxicity from cancer therapy. Furthermore, these criteria include specific cancer entities, among them ALL with Robertsonian translocation rob(15;21), hypodiploid ALL, AML with monosomy 7, and MDS in general. Other features are somatic hits suggesting a germline predisposition, more than two malignancies in the diseased child, a number of defined congenital defects or high/ unexpected toxicity [26].

The Nordic guidelines established by the Nordic MDS group initially focused on adults with germline predisposition to myeloid neoplasms and are intended for use in clinical practice [27]. They provide most detailed recommendations, based on the recent literature and clinical experience of the group, for the identification of individuals with genetic predisposition for hematological malignancies. These guidelines contain clear algorithms on who should be offered genetic testing based on age, family history, personal medical history, clinical/ physical findings and genetic characterization of the clonal cells, e.g. they identify patients below the age of 50 with monosomy 7, del(7q) or der(1;7) as possible candidates for familial leukemia, who should, therefore, be investigated for cancer predisposition genes. In clinical studies, it is recommended to investigate even *all* patients below 50 years of age (Table 1).

Since leukemia patients with genetic predisposition may need special clinical care, the 2017 ELN recommendations [11] have already stated the importance of identifying these patients. Affected patients, and their families, should be offered genetic counseling with a counselor familiar with these disorders. Recently, the Nordic MDS group has published the Nordic guidelines for germline predisposition to myeloid neoplasms in adults within an EHA guideline article. The Nordic guidelines contain very concise and clear recommendations for diagnostic work-up, genetic diagnosis, clinical management and follow-up. Most importantly, they see a close collaboration between hematologists and clinical geneticists as mandatory [27]. Genetic counseling should be offered to all patients investigated for potential germline conditions, even if no pathogenic variant is detected. Genetic counseling is highly recommended *prior* to genetic testing of all patients, (e.g. upon detection of a potential germline variant in clonal cells of an MDS/ AML patient), and also prior to genetic testing of apparently healthy relatives including predictive testing of HLA-identical potential family donors. The American Association for Cancer Research (AACR) also highly recommends referral to specialized centers with expertise in hereditary hematologic malignancies including hematologists-oncologists and geneticists to enable coordinated and comprehensive care [28]. Talking points to discuss with families include genetic risk, clinical management of hematologic and non-hematologic malignancies, screening, and participation in research programs. Moreover, the expert group found it important to educate the families about signs and symptoms of leukemia and to initiate an early consultation with transplant specialists. If genetic testing of siblings and parents is planned to exclude mutation carriers as stem cell donors, pre- and post-test genetic counselling should be provided [28].

## Identification of pathogenic germline variants in genes associated with predisposition to hematological malignancies

There is a scientific debate as to what tissue serves best as a source of germline DNA for hematopoietic malignancies. The Nordic MDS group recommends the use of skin fibroblasts and isolated T cells.

Simon et al*.* have suggested investigating buccal swaps or saliva obtained at diagnosis [29]. Pairwise comparison of this “normal DNA” with AML samples followed two criteria: (1) the suggested germline variant (e.g. *RUNX1*) should show a variant allele frequency of nearly 50% in the normal DNA, (2) in contrast to the germline variant, additional genetic markers such as *NRAS* should only be present in the AML sample but not in normal DNA [30–32]. Using this approach, 12 of 44 *RUNX1* pathogenic variants have been validated to be of germline origin [29]. Even though this approach holds undeniable advantages over the usage of bone marrow aspirates, the possibility of multiple clonal somatic variants with different allele frequencies still have to be kept in mind. In case of remission bone marrow aspirates or peripheral blood remain an option, provided that the methods necessary to measure any residual blast contamination are available.

It is a challenge to discriminate mosaicism, i.e. the presence of genetic variants in different organs and tissues developing due to post-zygotic events, from clonal hematopoiesis, i.e. somatic variants in genes known to be drivers of hematologic malignancy leading to an expansion of hematopoietic cells. [33, 34] If this question arises, not only peripheral blood or bone marrow, but additional tissues have to be investigated [35].

The Spanish guidelines for the use of targeted deep sequencing in MDS and CMML include a section about genetic predisposition to be considered for variants with a variant allele frequency close to 50% or 100%. These variants should be evaluated within each patient’s clinical and familial context [36]. When a germline variant is confirmed, referral of patients to professionals with expertise in cancer predisposing syndromes and in genetic counselling is recommended. In line with these recommendations, the Nordic guidelines recommend further investigating somatic variants near heterozygosity (variant allele frequency (VAF) 40–60%), near homozygosity (VAF above 90%) and specific types of variants such as truncating *DDX41* variants.

Genetic testing to unravel a pathogenic germline variant in a gene responsible for predisposition to hematological malignancies should follow established quality criteria [37]. Only validated and accredited methods should be used to detect SNVs and CNVs including noncoding regulatory regions (e.g. in the *GATA2* intron 4). If panel analyses are used for the detection of SNV, array-based methods such as arrayCGH are mandatory for the identification of large deletions. As mentioned, large deletions comprise a significant portion of pathogenic variants, particularly in the *RUNX1* gene [23, 38].

In recent years, synonymous mutations (nucleotide changes in exonic regions that do not alter amino acid sequence and are usually excluded from the analytic pipelines) have been gaining attention as potential pathogenic mutations. For example, in GATA2 deficiency they account for ~ 6% of all cases and were shown to cause RNA degradation as confirmed by RNA sequencing [39]. The authors proposed a cascade approach where RNA sequencing follows inconclusive DNA analysis in patients with high index of suspicion for a specific Mendelian disorder but without a known pathogenic mutation. Another study performed in a cohort of families undergoing evaluation for hereditary cancer found that RNA genetic testing improves the interpretation of genetic variants found by DNA sequencing [40]. Evidence from RNA testing clarified the interpretation of 88% (49/56) of inconclusive cases, reclassifying them either as clinically actionable or as benign.

The distinction between (likely) benign and (likely) pathogenic variants is often difficult. According to the internationally accepted ACMG recommendations Richards et al*.* [41] have described benign variants as class 1, likely benign variants as class 2, VUS as class 3, likely pathogenic variants as class 4 and pathogenic variants as class 5. Criteria to determine the pathogenicity of a variant include cosegregation within the family, affecting a highly conserved region, disrupting a functionally important protein domain, complete loss of the gene, generation of a new slice site inducing exon skipping and a population frequency < 0.0001. For in silico prediction of disease-causing effects evolutionary conservation, functionally important protein domains, potential splice effects, conformational changes, homo-/heterozygosity, cis/trans, and familial segregation are considered. However, definitive proof for pathogenicity often requires functional studies. In the meantime, specific recommendations for the interpretation of *TP53* and *RUNX1* variants have been published [42, 43].

Since many families with hereditary cancers have unique and often novel mutations, a substantial number of variants identified are VUS. If a genetic alteration has been classified as VUS, it cannot be included in a clinical report as having clinical significance. However, insights from experts (who might know of additional patients with identical mutations, not previously published or deposited in variant databases such as ClinVar) might help reclassifying such VUS changes. Therefore, interdisciplinary variant interpretation boards or consortia will be key to improved classification of germline variants. A VUS may be included in a clinical report for future reference in case that it is reclassified and becomes clinically relevant. Until a VUS is successfully classified, however, there should be no clinical significance resulting from it. It has to be noted that extensive research and publication of data are needed before a VUS may become clinically relevant.

## Recommendations for screening of patients with genetic predisposition for hematologic malignancies

The screening program is recommended only for patients with (likely) pathogenic variants, but not for variants of unknown significance and should also be offered to family members at risk [27].

According to the recent recommendations of the AACR [28], there is no benefit of screening in rapidly evolving leukemia such as AML or ALL. However, screening may be helpful for timing of allo-stem cell transplantation in some bone marrow failure syndromes predisposing for AML or MDS.

AACR recommends an annual surveillance testing with complete blood count (CBC) and bone marrow (BM) aspirate/biopsies with cytogenetics. At baseline CBC with differential, BM aspirate/biopsy with cytogenetics and a myeloid panel is recommended to investigate somatic variants. A CBC as a follow-up should be performed every six months for patients and family members at risk that carry RUNX1, GATA2 or Fanconi anemia pathogenic variants. Only in case of worsening CBC, BM aspirate/biopsy with cytogenetics and a myeloid genetic panel should be performed. An innovative recommendation is to do an annual blood testing for somatic pathogenic variants to follow clonal evolution [27]. Clonal hematopoiesis may be used to determine the time point for early intervention. These data should be evaluated in the context of the type and number of variants, the gene involved, the variant allele frequency (VAF), and also the dynamics over time.

It is also important to consider other organ manifestations such as the high risk for early breast cancer in females with Li-Fraumeni syndrome. Thus, children with hypodiploid ALL carrying pathogenic variants of *TP53*, should be included in intensive screening programs that have demonstrated improved outcome [44–47]. Whenever possible, avoiding exogenic noxes such as repeated radiography and radiotherapy may help to decrease the risk of secondary cancer in Li-Fraumeni syndrome and other DNA repair deficiency syndromes.

## Conclusion

Inherited genetic variants may tailor treatment and screening aiming at early intervention. The knowledge of inherited genetic variants may also be important for transplant planning (e.g. to exclude family members that also carry the genetic variant as possible donors).

In essence, expert centers with close collaboration of hemato-oncologist and clinical geneticists are needed to guide diagnosis, treatment and screening. Expert genetic counselling is essential to inform family members at risk and guide screening.

Statements regarding patients and healthy family members carrying pathogenic germline variants predisposing them to hematologic malignancies

- Awareness for the clinical features of inherited forms of leukemia should be raised to identify patients in need of special care.
- Clinical care should be provided by an interdisciplinary team of hematologists and clinical geneticists familiar with this type of diseases.
- Expert genetic counselling should be offered to patients and healthy family members at risk.
- Genetic analyses should follow established quality criteria, including the determination of pathogenicity of variants and clinical reporting.
- Screening should be performed in the context of clinical studies.
- International registers to collect clinical, genetic and outcome data are urgently needed.
- Genetic data, particularly VUS, and associated phenotypes should be shared among diagnostic laboratories.

## References

[1] Churpek JE, Pyrtel K, Kanchi KL, Shao J, Koboldt D, Miller CA, Shen D, Fulton R, O'Laughlin M, Fronick C, Pusic I, Uy GL, Braunstein EM, Levis M, Ross J, Elliott K, Heath S, Jiang A, Westervelt P, DiPersio JF, Link DC, Walter MJ, Welch J, Wilson R, Ley TJ, Godley LA, Graubert TA (2015). Genomic analysis of germ line and somatic variants in familial myelodysplasia/acute myeloid leukemia. Blood.

[2] Wlodarski MW, Hirabayashi S, Pastor V, Stary J, Hasle H, Masetti R, Dworzak M, Schmugge M, van den Heuvel-Eibrink M, Ussowicz M, De Moerloose B, Catala A, Smith OP, Sedlacek P, Lankester AC, Zecca M, Bordon V, Matthes-Martin S, Abrahamsson J, Kuhl JS, Sykora KW, Albert MH, Przychodzien B, Maciejewski JP, Schwarz S, Gohring G, Schlegelberger B, Cseh A, Noellke P, Yoshimi A, Locatelli F, Baumann I, Strahm B, Niemeyer CM, Ewog MDS (2016). Prevalence, clinical characteristics, and prognosis of GATA2-related myelodysplastic syndromes in children and adolescents. Blood.

[3] Wlodarski MW, Niemeyer CM (2017). Introduction: genetic syndromes predisposing to myeloid neoplasia. Semin Hematol.

[4] Owen C, Fitzgibbon J (2010) The genetics of acute myeloid leukemias, vol 3rd edn. Molecular Hematology. 10.1002/9781444318531.ch4

[5] Ripperger T, Steinemann D, Gohring G, Finke J, Niemeyer CM, Strahm B, Schlegelberger B (2009). A novel pedigree with heterozygous germline RUNX1 mutation causing familial MDS-related AML: can these families serve as a multistep model for leukemic transformation?. Leukemia.

[6] Walsh MF, Chang VY, Kohlmann WK, Scott HS, Cunniff C, Bourdeaut F, Molenaar JJ, Porter CC, Sandlund JT, Plon SE, Wang LL, Savage SA (2017). Recommendations for childhood cancer screening and surveillance in DNA repair disorders. Clin Cancer Res.

[7] Savage SA, Cook EF (2015) Dyskeratosis congenita and telomere biology disorders: diagnosis and management guidelines. These guidelines are posted as a PDF at wwwdcoutreachorg

[8] Dror Y, Donadieu J, Koglmeier J, Dodge J, Toiviainen-Salo S, Makitie O, Kerr E, Zeidler C, Shimamura A, Shah N, Cipolli M, Kuijpers T, Durie P, Rommens J, Siderius L, Liu JM (2011). Draft consensus guidelines for diagnosis and treatment of Shwachman-Diamond syndrome. Ann Ny Acad Sci.

[9] Hays L, Frohnmayer D, Frohnmayer L, Guinan E, Kennedy T, Larsen K (2014) Fanconi Anemia: guidelines for diagnosis and management, 4th (edn). wwwfanconiorg

[10] Arber DA, Orazi A, Hasserjian R, Thiele J, Borowitz MJ, Le Beau MM, Bloomfield CD, Cazzola M, Vardiman JW (2016). The 2016 revision to the World Health Organization classification of myeloid neoplasms and acute leukemia. Blood.

[11] Dohner H, Estey E, Grimwade D, Amadori S, Appelbaum FR, Buchner T, Dombret H, Ebert BL, Fenaux P, Larson RA, Levine RL, Lo-Coco F, Naoe T, Niederwieser D, Ossenkoppele GJ, Sanz M, Sierra J, Tallman MS, Tien HF, Wei AH, Lowenberg B, Bloomfield CD (2017). Diagnosis and management of AML in adults: 2017 ELN recommendations from an international expert panel. Blood.

[12] Pastor VB, Sahoo SS, Boklan J, Schwabe GC, Saribeyoglu E, Strahm B, Lebrecht D, Voss M, Bryceson YT, Erlacher M, Ehninger G, Niewisch M, Schlegelberger B, Baumann I, Achermann JC, Shimamura A, Hochrein J, Tedgard U, Nilsson L, Hasle H, Boerries M, Busch H, Niemeyer CM, Wlodarski MW (2018). Constitutional SAMD9L mutations cause familial myelodysplastic syndrome and transient monosomy 7. Haematologica.

[13] Fischer U, Yang JJ, Ikawa T, Hein D, Vicente-Duenas C, Borkhardt A, Sanchez-Garcia I (2020). Cell Fate decisions: the role of transcription factors in early B-cell development and leukemia. Blood Cancer Discov.

[14] Gocho Y, Yang JJ (2019). Genetic defects in hematopoietic transcription factors and predisposition to acute lymphoblastic leukemia. Blood.

[15] Schlegelberger B, Kreipe H, Lehmann U, Steinemann D, Ripperger T, Gohring G, Thomay K, Rump A, Di Donato N, Suttorp M (2015). A child with Li-Fraumeni syndrome: modes to inactivate the second allele of TP53 in three different malignancies. Pediatr Blood Cancer.

[16] Schlegelberger B, Heller PG (2017). RUNX1 deficiency familial platelet disorder with predisposition to myeloid leukemia, FPDMM. Semin Hematol.

[17] Feurstein S, Godley LA (2017). Germline ETV6 mutations and predisposition to hematological malignancies. Int J Hematol.

[18] Auer F, Ruschendorf F, Gombert M, Husemann P, Ginzel S, Izraeli S, Harit M, Weintraub M, Weinstein OY, Lerer I, Stepensky P, Borkhardt A, Hauer J (2014). Inherited susceptibility to pre B-ALL caused by germline transmission of PAX5 c.547G>A. Leukemia.

[19] Duployez N, Jamrog LA, Fregona V, Hamelle C, Fenwarth L, Lejeune S, Helevaut N, Geffroy S, Caillault A, Marceau-Renaut A, Poulain S, Roche-Lestienne C, Largeaud L, Prade N, Dufrechou S, Hébrard S, Berthon C, Nelken B, Fernandes J, Villenet C, Figeac M, Gerby B, Delabesse E, Preudhomme C, Broccardo C (2021). Germline PAX5 mutation predisposes to familial B-cell precursor acute lymphoblastic leukemia. Blood.

[20] Shah S, Schrader KA, Waanders E, Timms AE, Vijai J, Miething C, Wechsler J, Yang J, Hayes J, Klein RJ, Zhang J, Wei L, Wu G, Rusch M, Nagahawatte P, Ma J, Chen SC, Song G, Cheng J, Meyers P, Bhojwani D, Jhanwar S, Maslak P, Fleisher M, Littman J, Offit L, Rau-Murthy R, Fleischut MH, Corines M, Murali R, Gao X, Manschreck C, Kitzing T, Murty VV, Raimondi S, Kuiper RP, Simons A, Schiffman JD, Onel K, Plon SE, Wheeler D, Ritter D, Ziegler DS, Tucker K, Sutton R, Chenevix-Trench G, Li J, Huntsman DG, Hansford S, Senz J, Walsh T, Lee M, Hahn CN, Roberts K, King MC, Lo SM, Levine RL, Viale A, Socci ND, Nathanson KL, Scott HS, Daly M, Lipkin SM, Lowe SW, Downing JR, Altshuler D, Sandlund JT, Horwitz MS, Mullighan CG, Offit K (2013). A recurrent germline PAX5 mutation confers susceptibility to pre-B cell acute lymphoblastic leukemia. Nat Genet.

[21] Churchman ML, Qian M, Te Kronnie G, Zhang R, Yang W, Zhang H, Lana T, Tedrick P, Baskin R, Verbist K, Peters JL, Devidas M, Larsen E, Moore IM, Gu Z, Qu C, Yoshihara H, Porter SN, Pruett-Miller SM, Wu G, Raetz E, Martin PL, Bowman WP, Winick N, Mardis E, Fulton R, Stanulla M, Evans WE, Relling MV, Pui CH, Hunger SP, Loh ML, Handgretinger R, Nichols KE, Yang JJ, Mullighan CG (2018). Germline genetic IKZF1 variation and predisposition to childhood acute lymphoblastic leukemia. Cancer Cell.

[22] Vicente-Duenas C, Janssen S, Oldenburg M, Auer F, Gonzalez-Herrero I, Casado-Garcia A, Isidro-Hernandez M, Raboso-Gallego J, Westhoff P, Pandyra AA (2020). An intact gut microbiome protects genetically predisposed mice against leukemia. Blood.

[23] Rio-Machin A, Vulliamy T, Hug N, Walne A, Tawana K, Cardoso S, Ellison A, Pontikos N, Wang J, Tummala H, Al Seraihi AFH, Alnajar J, Bewicke-Copley F, Armes H, Barnett M, Bloor A, Bodor C, Bowen D, Fenaux P, Green A, Hallahan A, Hjorth-Hansen H, Hossain U, Killick S, Lawson S, Layton M, Male AM, Marsh J, Mehta P, Mous R, Nomdedeu JF, Owen C, Pavlu J, Payne EM, Protheroe RE, Preudhomme C, Pujol-Moix N, Renneville A, Russell N, Saggar A, Sciuccati G, Taussig D, Toze CL, Uyttebroeck A, Vandenberghe P, Schlegelberger B, Ripperger T, Steinemann D, Wu J, Mason J, Page P, Akiki S, Reay K, Cavenagh JD, Plagnol V, Caceres JF, Fitzgibbon J, Dokal I (2020). The complex genetic landscape of familial MDS and AML reveals pathogenic germline variants. Nat Commun.

[24] Ripperger T, Tauscher M, Haase D, Griesinger F, Schlegelberger B, Steinemann D (2011). Managing individuals with propensity to myeloid malignancies due to germline RUNX1 deficiency. Haematologica.

[25] Jongmans MC, Loeffen JL, Waanders E, Hoogerbrugge PM, Ligtenberg MJ, Kuiper RP, Hoogerbrugge N (2016). Recognition of genetic predisposition in pediatric cancer patients: an easy-to-use selection tool. Eur J Med Genet.

[26] Ripperger T, Bielack SS, Borkhardt A, Brecht IB, Burkhardt B, Calaminus G, Debatin KM, Deubzer H, Dirksen U, Eckert C, Eggert A, Erlacher M, Fleischhack G, Fruhwald MC, Gnekow A, Goehring G, Graf N, Hanenberg H, Hauer J, Hero B, Hettmer S, von Hoff K, Horstmann M, Hoyer J, Illig T, Kaatsch P, Kappler R, Kerl K, Klingebiel T, Kontny U, Kordes U, Korholz D, Koscielniak E, Kramm CM, Kuhlen M, Kulozik AE, Lamottke B, Leuschner I, Lohmann DR, Meinhardt A, Metzler M, Meyer LH, Moser O, Nathrath M, Niemeyer CM, Nustede R, Pajtler KW, Paret C, Rasche M, Reinhardt D, Riess O, Russo A, Rutkowski S, Schlegelberger B, Schneider D, Schneppenheim R, Schrappe M, Schroeder C, von Schweinitz D, Simon T, Sparber-Sauer M, Spix C, Stanulla M, Steinemann D, Strahm B, Temming P, Thomay K, von Bueren AO, Vorwerk P, Witt O, Wlodarski M, Wossmann W, Zenker M, Zimmermann S, Pfister SM, Kratz CP (2017). Childhood cancer predisposition syndromes-A concise review and recommendations by the **cancer predisposition working group of the society for pediatric oncology and hema**tology. Am J Med Genet A.

[27] Baliakas P, Tesi B, Wartiovaara-Kautto U, Stray-Pedersen AR, Friis LS, Dybedal I, Hovland R, Jahnukainen K, Raaschou-Jensen K, Ljungman P, Rustad CF, Lautrup CK, Kilpivaara O, Kittang AO, Gr Nbaek K, Cammenga J, Hellstrom-Lindberg E, Andersen MK (2019). Nordic guidelines for germline predisposition to myeloid neoplasms in adults: recommendations for genetic diagnosis. Clinical Management and Follow-up Hemasphere.

[28] Porter CC, Druley TE, Erez A, Kuiper RP, Onel K, Schiffman JD, Wolfe Schneider K, Scollon SR, Scott HS, Strong LC, Walsh MF, Nichols KE (2017). Recommendations for surveillance for children with leukemia-predisposing conditions. Clin Cancer Res.

[29] Simon L, Spinella JF, Yao CY, Lavallee VP, Boivin I, Boucher G, Audemard E, Bordeleau ME, Lemieux S, Hebert J, Sauvageau G (2020). High frequency of germline RUNX1 mutations in patients with RUNX1-mutated AML. Blood.

[30] Godley LA, Shimamura A (2017). Genetic predisposition to hematologic malignancies: management and surveillance. Blood.

[31] Godley LA (2014). Inherited predisposition to acute myeloid leukemia. Semin Hematol.

[32] Brown AL, Churpek JE, Malcovati L, Dohner H, Godley LA (2017). Recognition of familial myeloid neoplasia in adults. Semin Hematol.

[33] Boettcher S, Ebert BL (2019). Clonal hematopoiesis of indeterminate potentia. JCO.

[34] Jasiwal S, Ebert BL (2019). Clonal hematopoiesis in human aging and disease. Science.

[35] Golas MM, Auber B, Ripperger T, Pabst B, Schmidt G, Morlot M, Diebold U, Steinemann D, Schlegelberger B, Morlot S (2019). Looking for the hidden mutation: Bannayan–Riley–Ruvalcaba syndrome caused by constitutional and mosaic 10q23 microdeletions involving PTEN and BMPR1A. AJMG.

[36] Palomo L, Ibanez M, Abaigar M, Vazquez I, Alvarez S, Cabezon M, Tazon-Vega B, Rapado I, Fuster-Tormo F, Cervera J, Benito R, Larrayoz MJ, Cigudosa JC, Zamora L, Valcarcel D, Cedena MT, Acha P, Hernandez-Sanchez JM, Fernandez-Mercado M, Sanz G, Hernandez-Rivas JM, Calasanz MJ, Sole F, Such E (2020). Spanish guidelines for the use of targeted deep sequencing in myelodysplastic syndromes and chronic myelomonocytic leukaemia. Brit J Haematol.

[37] Humangenetiker BD (2018). Sk-leitlinie humangenetische diagnostik und genetische beratung. Medgen.

[38] Ripperger T, Tawana K, Kratz C, Schlegelberger B, Fitzgibbon J, Steinemann D (2016). Clinical utility gene card for: familial platelet disorder with associated myeloid malignancies. Eur J Hum Genet.

[39] Kozyra EJ, Pastor VB, Lefkopoulos S, Sahoo SS, Busch H, Voss RK, Erlacher M, Lebrecht D, Szvetnik EA, Hirabayashi S, Pasauliene R, Pedace L, Tartaglia M, Klemann C, Metzger P, Boerries M, Catala A, Hasle H, de Haas V, Kallay K, Masetti R, De Moerloose B, Dworzak M, Schmugge M, Smith O, Stary J, Mejstrikova E, Ussowicz M, Morris E, Singh P, Collin M, Derecka M, Göhring G, Flotho C, Strahm B, Locatelli F, Niemeyer CM, Trompouki E, Wlodarski MW (2020). Synonymous GATA2 mutations result in selective loss of mutated RNA and are common in patients with GATA2 deficiency. Leukemia.

[40] Karam R, Conner B, LaDuca H, McGoldrick K, Krempely K, Richardson ME, Zimmermann H, Gutierrez S, Reineke P, Hoang L, Allen K, Yussuf A, Farber-Katz S, Rana HQ, Culver S, Lee J, Nashed S, Toppmeyer D, Collins D, Haynes G, Pesaran T, Dolinsky JS, Tippin Davis B, Elliott A, Chao E (2019). Assessment of diagnostic outcomes of RNA genetic testing for hereditary cancer. JAMA Netw Open.

[41] Richards S, Aziz N, Bale S, Bick D, Das S, Gastier-Foster J, Grody WW, Hegde M, Lyon E, Spector E, Voelkerding K, Rehm HL, Committee ALQA (2015). Standards and guidelines for the interpretation of sequence variants: a joint consensus recommendation of the American college of medical genetics and genomics and the association for molecular pathology. Genet Med.

[42] Fortuno C, Lee K, Olivier M, Pesaran T, Mai PL, de Andrade KC, Attardi LD, Crowley S, Evans DG, Feng BJ, Foreman AKM, Frone MN, Huether R, James PA, McGoldrick K, Mester J, Seifert BA, Slavin TP, Witkowski L, Zhang L, Plon SE, Spurdle AB, Savage SA (2021). ClinGen TP53 variant curation expert panel specifications of the ACMG/AMP variant interpretation guidelines for germline TP53 variants. Hum Mutat.

[43] Luo X, Feurstein S, Mohan S, Porter CC, Jackson SA, Keel S, Chicka M, Brown AL, Kesserwan C, Agarwal A, Luo M, Li Z, Ross JE, Baliakas P, Pineda-Alvarez D, DiNardo CD, Bertuch AA, Mehta N, Vulliamy T, Wang Y, Nichols KE, Malcovati L, Walsh MF, Rawlings LH, McWeeney SK, Soulier J, Raimbault A, Routbort MJ, Zhang L, Ryan G, Speck NA, Plon SE, Wu D, Godley LA (2019). ClinGen myeloid malignancy variant curation expert panel recommendations for germline RUNX1 variants. Blood Adv.

[44] Malard F, Mohty M (2020). Acute lymphoblastic leukaemia. Lancet.

[45] Frebourg T, Bajalica Lagercrantz S, Oliveira C, Magenheim R, Evans DG, European Reference Network (2020). Guidelines for the Li-Fraumeni and heritable TP53-related cancer syndromes. Eur J Hum Genet.

[46] Kratz CP, Achatz MI, Brugieres L, Frebourg T, Garber JE, Greer MC, Hansford JR, Janeway KA, Kohlmann WK, McGee R, Mullighan CG, Onel K, Pajtler KW, Pfister SM, Savage SA, Schiffman JD, Schneider KA, Strong LC, Evans DGR, Wasserman JD, Villani A, Malkin D (2017). Cancer screening recommendations for individuals with Li-Fraumeni syndrome. Clin Cancer Res.

[47] Kratz CP, Villani A, Nichols KE, Schiffman J, Malkin D (2020). Cancer surveillance for individuals with Li-Fraumeni syndrome. Eur J Hum Genet.

